# Aminoglycosides for the treatment of septic shock: a propensity-based study

**DOI:** 10.1186/s13054-020-03284-9

**Published:** 2020-09-21

**Authors:** Jean-François Llitjos, Simon Meslin, Swann Bredin, Matthieu Jamme, Frédéric Pène

**Affiliations:** 1 Service de médecine intensive-réanimation, Hôpital Cochin, Hôpitaux Universitaires Paris-Centre, Assistance Publique - Hôpitaux de Paris, 27 rue du Faubourg Saint-Jacques, 75014 Paris, France; 2 Université de Paris, Paris, France; 3 Institut Cochin, INSERM U1016, CNRS UMR 8104, Paris, France; 4 Réanimation médico-chirurgicale, hôpital Poissy Saint-Germain, Poissy, France; 5 INSERM U-1018, CESP, Team 5 (EpReC, Renal and Cardiovascular Epidemiology), Université Versailles Saint-Quentin, Villejuif, France

**Keywords:** Septic shock, Aminoglycosides, Antibiotics, Intensive care unit, Bacterial resistance, Bacteria

The adequacy of initial antimicrobial treatment is a strong determinant of prognosis in septic shock. The prototypic synergistic combination of beta-lactams with aminoglycosides appears as an attractive therapeutic option, but its actual benefit remains elusive [1, 2]. We took advantage of a large comprehensive cohort of septic shock to address the impact of aminoglycosides on mortality, with respect to their pharmacodynamic and pharmacokinetic properties.

We performed a retrospective single-center study over a 9-year period (2008–2016) of patients admitted to the intensive care unit (ICU) for septic shock, defined as microbiologically proven or clinically suspected infection associated with acute circulatory failure requiring vasopressors. The primary endpoint was in-ICU mortality. Patients treated or non-treated with aminoglycosides were matched in a 1:1 ratio using a logistic regression-based propensity score including the following variables: age, gender, comorbid conditions, SAPS2, source of infection, biological findings, and organ supports at admission. Accuracy of aminoglycoside administration was characterized by the loading dose (recommended as 30 mg/kg amikacin or 6 mg/kg gentamycin/tobramycin) and the peak serum concentration (C_peak_) (targets recommended as ≥ 60 mg/L amikacin or ≥ 30 mg/L gentamicin/tobramycin). Determinants of mortality were investigated in cause-specific proportional hazard model.

Among the 1040 patients, 616 (59%) were administered a primary antibiotic combination regimen of beta-lactam with amikacin (379 patients, 62%), gentamycin (229 patients, 37%), or tobramycin (8 patients, 1%). The overall mortality rate was 35%. The propensity score-based matching process resulted in two cohorts of 348 patients with and without aminoglycosides (Table 1). Using the SAPS-2 score, the severity was comparable between the two groups after matching (68 points (52–85) in the aminoglycoside group versus 65 points (51–80) in the non-aminoglycoside group (*p* = 0.17)). Among patients with microbiologically documented infections, the adequacy of the initial antibiotic regimen increased from 82% with single beta-lactam to 92% with combination regimen (*p* = 0.01). In combination-treated patients, 74% of documented pathogens were susceptible to both antibiotics whereas 12% were only susceptible to aminoglycosides. Loading doses of the first aminoglycoside infusion were appropriate in 21% of amikacin-treated and 27% of gentamycin/tobramycin-treated patients. Hence, only 18% of patients with available C_peak_ measurements achieved recommended concentration targets (30% for amikacin while none for gentamycin/tobramycin) (Fig. 1). Furthermore, it is important to take into account that pneumonia is the main source of septic shock treated with aminoglycosides whereas their diffusion is poor in lung tissue.

**Table 1:** Characteristics of septic shock patients treated or not with aminoglycosides before and after matching on the propensity score

Variables	Before matching		***p***	After matching		***p***
	Aminoglycosides (***n*** = 616)	No aminoglycosides (***n*** = 424)		Aminoglycosides (***n*** = 348)	No aminoglycosides (n = 348)	
Age, years	**70.2 (58.2–79.7)**	**67.5 (56.5–77.5)**	**0.02**	**70.2 (56.6–79)**	**66.9 (55.9–76.9)**	**0.06**
Male gender	**386 (62)**	**278 (65)**	**0.36**	**227 (65)**	**228 (65)**	**1**
Immunosuppression	**249 (40)**	**135 (31)**	**0.005**	**127 (37)**	**105 (30)**	**0.09**
Neutropenia	**91 (15)**	**38 (9)**	**0.006**	**41 (12)**	**30 (9)**	**0.21**
Characteristics on ICU admission						
SAPS2, points	**70 (53–87)**	**65 (51–80)**	**0.002**	**68 (52–85)**	**65 (51–80)**	**0.17**
Source of infection						**0.29**
Lung	**241 (39)**	**248 (58)**	**< 0.001**	**169 (48)**	**202 (58)**	
Digestive	**82 (13)**	**46 (11)**		**44 (13)**	**37 (11)**	
Urinary	**101 (17)**	**24 (5)**		**24 (7)**	**17 (5)**	
Skin and soft tissue	**57 (9)**	**24 (5)**		**30 (9)**	**23 (7)**	
Catheter	**33 (5)**	**12 (3)**		**13 (4)**	**9 (3)**	
Others	**29 (5)**	**23 (5)**		**20 (6)**	**21 (6)**	
Unknown	**73 (12)**	**47 (11)**		**48 (14)**	**39 (12)**	
Microbiological documentation	**419 (68)**	**263 (62)**	**0.05**	**226 (65)**	**228 (66)**	**0.93**
Bacteremia	**241 (39)**	**106 (25)**	**< 0.001**	**114 (33)**	**90 (26)**	**0.055**
Microorganisms						
Gram-negative bacteria	**290 (47)**	**131 (31)**	**< 0.001**	**137 (60)**	**102 (45)**	**0.003**
Gram-positive bacteria	**120 (19)**	**120 (28)**		**85 (38)**	**114 (50)**	
Fungi	**9 (1)**	**10 (2)**		**4 (2)**	**11 (4.5)**	
Mycobacteria	**0 (0)**	**2 (0.5)**		**0 (0)**	**1 (0.5)**	
Biological findings						
Serum protein level, g/L	**56 (49–64)**	**60 (53–68)**	**< 0.001**	**58 (51–66)**	**60.5 (53–68)**	**0.009**
Serum creatinine level, μmol/L	**144 (95–228)**	**131 (80–201)**	**0.007**	**139 (84–225)**	**132 (82–206)**	**0.23**
ICU management at day 1						
First 24-h fluid balance, mL	**2485 (1000–4378)**	**2088 (717–3552)**	**< 0.001**	**2358 (900–4200)**	**2136 (900–3700)**	**0.18**
Renal replacement therapy at day 1	**110 (18)**	**59 (14)**	**0.13**	**58 (17)**	**52 (15)**	**0.60**
Norepinephrine amount at day 1, mg	**28.2 (8.5–73)**	**18.2 (5.3–48.2)**	**< 0.001**	**25 (5.8–55.2)**	**20 (6.9–50.3)**	**0.20**
Aminoglycosides treatment						
Administration prior to ICU admission	116 (19)			50 (14)		
Amikacin	**379 (62)**			**218 (63)**		
Loading dose, mg/kg	19.7 (17.2–23.6)			20 (17–24)		
Recommended loading dose	78 (21)			51 (23)		
Median C_peak_, mg/L *	52.4 (34.8–61)			47.2 (35.7–60.6)		
Recommended target	36 (33)			18 (30)		
Gentamicin/Tobramycin	**237 (38)**			**130 (37)**		
Loading dose, mg/kg	4.7 (4.2–6.1)			5.4 (4.2–6)		
Recommended loading dose	63 (27)			40 (30)		
Median C_peak_, mg/L*	15.2 (10.6–19.9)			14.7 (10.4–19.6)		
Recommended target	4 (6)			0 (0)		
Overall ICU management						
Invasive mechanical ventilation	**500 (81)**	**366 (86)**	**0.03**	**292 (84)**	**305 (88)**	**0.19**
Renal replacement therapy	**319 (52)**	**162 (38)**	**< 0.001**	**179 (51)**	**131 (38)**	**< 0.001**
Outcomes						
Creatininemia at day 3, μmol/L	**85 (50–153)**	**85 (51–152.2)**	**0.71**	**91 (54–158.5)**	**89.5 (54.75–158.2)**	**0.86**
Creatininemia at discharge, μmol/L	**90 (58–173)**	**83 (54.75–144.2)**	**0.019**	**92.5 (58–177)**	**79.5 (54–137.5)**	**0.024**
End-of-life decision	**136 (22)**	**117 (28)**	**0.047**	**52 (15)**	**69 (20)**	**0.11**
7-day mortality	**142 (23)**	**75 (18)**	**0.03**	**65 (19)**	**51 (15)**	**0.12**
In-ICU mortality	**229 (37)**	**140 (33)**	**0.19**	**126 (36)**	**103 (29)**	**0.076**

*Among patients with available C_peak_; before matching: amikacin: 108 (28%) patients and gentamycin/tobramycin: 65 (27%) patients and after matching: amikacin: 60 (27%) patients and gentamycin/tobramycin: 38 (29%) patients

**Fig. 1:**
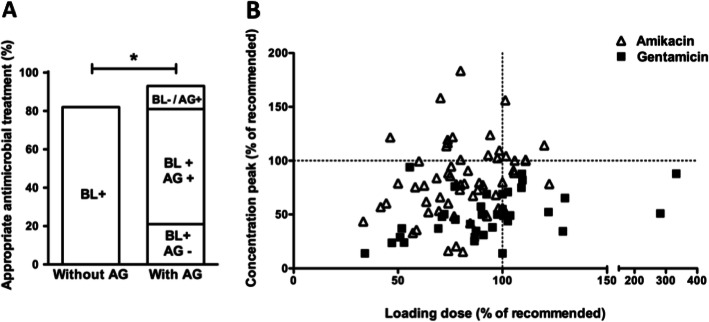
Pharmacodynamic and pharmacokinetic parameters associated with aminoglycoside treatment. **a** Adequacy of antimicrobial treatment among propensity score-matched patients with microbiologically documented infections treated or not with aminoglycosides BL+: beta-lactam efficient, BL-: beta-lactam non efficient, AG-: aminoglycosides non efficient and AG+: aminoglycosides efficient. *p = 0.01. **b** Relation between loading doses of aminoglycosides and concentration peak (C_peak_) among propensity score-matched patients. Loading doses and C_peak_ are expressed in percentage of recommended values. The horizontal line represents the value of the recommended C_peak_ for each aminoglycoside, and the vertical line depicts the value of the recommended loading dose

Aminoglycoside treatment was associated with worse outcomes, including increased requirements for renal replacement therapy during the ICU stay and higher creatinine levels at the time of ICU discharge, and trend towards increased in-ICU mortality (Table 1). Mortality rates of aminoglycoside-treated and aminoglycoside-untreated patients with microbiologically documented infections were not different (34% and 31%, respectively). In aminoglycoside patients who achieved the target concentration peak, the mortality was 28% whereas it was 33% in patients who did not (*p* = 0.76). After multivariate adjustment, aminoglycoside treatment was no longer associated with mortality (CSH 1.1; 95%CI 0.90–1.55, *p* = 0.25). Furthermore, aminoglycoside treatment did not impact on mortality in the relevant subgroups of neutropenic or bacteremic patients (CSH 1.11; 95%CI 0.75–1.62, *p* = 0.61 and CSH 1.03; 95%CI 0.64–1.66, *p* = 0.91, respectively).

Aminoglycosides harbor potent antimicrobial properties including bactericidal activity, synergy with beta-lactams, post-antibiotic effect, and broadening the antibacterial spectrum [3]. However, the evidence of benefit in septic shock is scarce, based on controversial meta-analysis and retrospective studies [1, 2, 4]. Despite the combination antibiotherapy improved the adequacy of initial antibiotic treatment, it did not translate into improved survival. However, the high incidence of aminoglycosides underdosing argues for accurate antimicrobial drug monitoring in further interventional trials [5].

## Data Availability

Yes
